# The ALSPAC Coordination Test (subtests of the Movement ABC): Methodology and data on associations with prenatal exposures to lead, cadmium and mercury

**DOI:** 10.1016/j.dib.2018.04.130

**Published:** 2018-05-05

**Authors:** Caroline M. Taylor, Alan M. Emond, Raghu Lingam, Jean Golding

**Affiliations:** a Centre for Child and Adolescent Health, Bristol Medical School, University of Bristol, UK; b School of Women's and Children's Health, UNSW Sydney, Australia

## Abstract

As part of the Avon Longitudinal Study of Parents and Children (ALSPAC), measures of child motor skills were collected in research clinics. The ALSPAC Coordination Test was derived from subtests of the Movement ABC at age 7 years in children participating in ALSPAC. Maternal blood lead, cadmium and mercury concentrations were measured by inductively-coupled plasma mass spectrometry in samples from women at a median gestation age of 11 weeks. Maternal reports at 32 weeks’ gestation were used to generate data on a range of potential confounders. The data were used to determine the associations between prenatal exposure to lead, cadmium and mercury and child motor skills at age 7 years. For results, please see Taylor et al. [1].

## Specifications Table

**Table:** 

Subject area	*Biology*
More specific subject area	*Child development*
Type of data	*Text and Tables*
How data was acquired	*Longitudinal cohort study questionnaire data, biological assessment*
Data format	*Edited and analysed*
Experimental factors	*Maternal self-completion questionnaires; maternal blood assays for lead, cadmium and mercury; clinic assessments of motor skills*
Experimental features	*Subtests scores from the Movement ABC test at 7 years in association with prenatal blood lead, cadmium and mercury concentrations levels*
Data source location	*Former Avon area, centred around Bristol, UK*
Data accessibility	*Data are within this article*

## Value of the data

• The ALSPAC dataset contains information on a large number of children in a geographically defined population whose development was monitored to age 25–26 years old at present (2018).
• The data provide a basis for early identification of adverse effects of environmental exposures (metals and other toxicants).
• The data allow detailed analyses of family and social circumstances and their associations with child development.

## 1 Data

In this paper, we describe data acquisition of scores from subtests of the Movement ABC at 7 years. We also include data from models of associations with prenatal blood lead, cadmium and mercury concentrations to support the main analyses in our parallel paper [1].

The ALSPAC study website contains details of all the data that are available through a fully searchable data dictionary: http://www.bris.ac.uk/alspac/researchers/data-access/data-dictionary/. Data can be obtained by bona fide researchers after application to the ALSPAC Executive Committee (http://www.bristol.ac.uk/alspac/researchers/access/).

## 2 Experimental design, materials and methods

### 2.1 Prenatal blood measurements of lead, cadmium and mercury

Sample collection and measurement of lead and mercury have been described in detail elsewhere [2], [3]. Cadmium concentrations in whole blood were measured similarly using inductively-coupled plasma mass spectrometry in standard mode (R. Jones, Centers for Disease Control and Prevention (CDC), Bethesda, MD, USA; CDC Method 3009.1) with appropriate quality controls.

### 2.2 Child measures of motor skills

Child motor skills were assessed at the age of 7 years by Movement ABC subtests of manual dexterity, ball skills and balance [4].

#### 2.2.1 Balance – heel to toe steps subtest

The procedure for this test has been described previously [5]. In brief, the child was asked to walk along a straight line (a line taped to the floor) without leaving any gaps of any size between the heel and the toe and without stepping off the line. The tester demonstrated the task first and emphasised these points. The child then had a practice of five steps. If the child made any procedural errors during the practice, the tester interrupted and reminded him or her of what to do. The child then began the main trial consisting of 15 steps along the line. The total number of successful steps out of a maximum of 15 was recorded.

#### 2.2.2 Ball skills – beanbag subtest

The procedure for this test has been described previously [6]. In brief, the child attempted to throw a beanbag into a box while standing behind a line at a distance of 6 ft (1.83 m) from the box. During the demonstration and explanation, the tester emphasised to the child that he or she should use only one-handed, underarm throws and remain behind the line for each throw, standing in a good position for throwing. The children were given five practice throws where they were able to change hands but were encouraged to choose their preferred hand for the main trial. Any procedural errors made during the practice were corrected and the children were reminded of, or re-demonstrated, the correct procedure. Out of 10 throws, the number to successfully land in the box was recorded.

#### 2.2.3 Manual dexterity – placing pegs subtest

In this task the child had to insert 12 pegs, one at a time into a peg board, holding the board with one hand and inserting the pegs with the other, as quickly as possible. The task was carried out with the preferred and the non-preferred hand, after it had been described and demonstrated by the tester, and after a practice attempt with each hand. The time taken to complete the task was recorded for each hand. If the child failed to carry out the instructions, for example, picked up more than one peg at a time, or used both hands to place the pegs, he or she failed that trial and a second trial with the same hand was given. Most children passed the first trial (> 90% using their preferred hand and > 85% using non-preferred hand) and in these cases a second trial was not carried out. About 4–6% of children failed the first trial but went on to pass the second trial. About 1% of children failed the first and second trial (they may have an artificially quick time because they used a short cut, or an artificially long time because they did not understand the instructions) and are not included in the data. Some children received a query for a trial – this was not a failure according to the test specifications but they might have done something that resulted in an unrealistically slow time (for example, the child knocked a peg off the table and it rolled away, making it difficult to retrieve). If the first trial was queried, a second trial was allowed and the faster time was accepted. If both trials received queries, the time from the first trial was accepted. If one trial was queried and the other passed, the time from the faster trial was accepted. If one trial was queried and the other failed, the time from the queried trial was accepted.

#### 2.2.4 Manual dexterity – threading lace subtest

This test required the child to thread a lace though holes in a wooden board. The child was asked to thread the lace in and out of the holes (rather than going around the edge of the board), using each hole and ensuring that the lace was pulled through each hole so that sufficient lace was left to complete the threading. The task was demonstrated by the tester and the child was given a practice attempt of completing two holes in the board, where any procedural errors were corrected immediately by the tester. The child was expected to use only one hand for using the lace, with the other hand holding the board. The time taken to complete the task was recorded. If the child failed to carry out the instructions or made procedural errors (for example, missed a hole in the board to threaded around the edges of the board), a second trial (with the same hand) was administered.

Subtest results were categorised as follows: heel to toe subtest 15 steps completed (pass) vs < 15 steps completed (fail) [5]; beanbag subtest 4–10 throws accurate (pass) or 0–3 throws accurate (fail) (poor skills defined as < 1 SD from mean [6]); threading lace 9–21 s (pass) vs 22–105 s (fail) (based on median value); peg board preferred hand 15–22 s (pass) vs 23–46 s (fail) (based on median value); peg board non-preferred hand 15–25 s (pass) vs 35–62 s (fail) (based on median value).

### 2.3 Developmental Coordination Disorder

Children with probable Developmental Coordination Disorder (DCD) were identified by using DSM-IV criteria adapted for research by using the 2006 Leeds Consensus Statement as described by Lingam et al. [7], [8]. Children with probable DCD met all four DSM-IV criteria: poor motor coordination (criterion A) that significantly interferes with activities of daily living (ADL) or academic achievement (criterion B), with the disturbance neither being a result of a general medical condition nor meeting the criteria for a pervasive developmental disorder (criterion C), and inexplicable by intellectual disability (criterion D). The following exclusion criteria were applied to the data from the Movement ABC subtests: (1) known visual deficit or neurological condition such as cerebral palsy; (2) IQ < 70. Children with a diagnosis of autism spectrum disorder, identified from linked education and health records, were not excluded provided their IQ was ≥ 70 and they had no known visual deficit or neurological disorder. Those children who were < 15th centile on motor testing and in addition either failed their National Curriculum Key Stage 1 writing test and/or were < 15th centile on a 23-item ADL scale derived from parent-completed questionnaires were identified as having probable DCD (5% of the eligible ALSPAC cohort). The term ‘probable’ was used as these children did not have a clinical examination.

### 2.4 Questionnaire assessments

The ALSPAC study included the distribution of questionnaire by mail to the pregnant woman for self-completion and return in a pre-paid envelope at 32 weeks’ gestation.

### 2.5 Publications

Publications on child motor skills and on DCD in the ALSPAC cohort are shown in Table 1.

**Table 1:** Publications using measures of child motor skills in the ALSPAC cohort.

**Authors**	**Exposure**	**Outcome**	**Results**
Golding et al. [6]	Exposome approach using longitudinal data covering three generations	Balls skills from Movement ABC subtests at 7 years	Negative association with mother's unhappiness in her mid-childhood (6–11 years); the offspring of parents who had poor eyesight had poorer motor ability
Humphriss et al. [5]	Aim: to describe the balance test results at 7 years		Frequency distributions for each subtest
Taylor et al. [9]	Prenatal lead and cadmium exposure	Balance ability at 7 years	No evidence of associations of prenatal exposure to lead or cadmium with balance ability
Taylor et al. [1]	Prenatal lead, cadmium and mercury exposure	Subtests of the Movement ABC test (ALSPAC Co-ordination test)	No evidence of associations of prenatal exposure to lead, cadmium or mercury with any subtest of motor skills
Lingam et al. [8]	Aim: to describe prevalence of Developmental Coordination Disorder at 7 years		Prevalence 17 per 1000 children
Lingam et al. [7]	Developmental Coordination Disorder	Attention, language, social skills and academic ability	Increased risk of difficulties in attention, social skills, reading and spelling

### 2.6 Associations with prenatal lead, cadmium and mercury

In our parallel paper [1] we show there was no evidence to support a role of prenatal exposure to heavy metals at these levels on motor skills in the child at age 7 years measured using the Movement ABC. Here we show (Table 1, Table 2, Table 3, Table 4, Table 5, Table 6):

(i) Publications using measures of child motor skills in the ALSPAC cohort.
(ii) Associations of motor skills with prenatal blood lead, cadmium or mercury concentrations as continuous variables: complete cases (*n* = 1558).
(iii) Motor skills in children at age 7 years by category of prenatal lead, cadmium or mercury exposure: complete cases (*n* = 1558).
(iv) Motor skills in child at age 7 years by category of prenatal lead, cadmium or mercury exposure: all cases.
(v) Association of motor skills with prenatal blood lead, cadmium or mercury exposure: all cases.
(vi) Associations of motor skills with quartiles of prenatal blood lead, cadmium or mercury exposure.

**Table 2:** Associations of motor skills with prenatal blood lead, cadmium or mercury concentrations as continuous variables: complete cases (*n* = 1558).

		**Prenatal blood lead**		**Prenatal blood cadmium**		**Prenatal blood mercury**	
		**OR (95% CI)**	***p***	**OR (95% CI)**	***p***	**OR (95% CI)**	***p***
**Balance**	Heel to toe: ref 15 steps (vs 1–14)						
	Unadjusted	1.01 (0.94, 1.07)	0.849	0.81 (0.67, 0.97)	0.025	1.04 (0.95, 1.14)	0.345
	Adjusted	0.98 (0.92, 1.05)	0.609	0.70 (0.52, 0.94)	0.019	1.00 (0.91, 1.10)	0.966
**Ball skills**	Beanbag: ref 7–10 throws (vs 1–3)						
	Unadjusted	0.98 (0.89, 1.07)	0.577	0.99 (0.76, 1.29)	0.926	0.93 (0.82, 1.04)	0.209
	Adjusted	0.98 (0.90, 1.08)	0.743	0.88 (0.60, 1.31)	0.536	0.93 (0.82, 1.06)	0.830
**Manual dexterity**	Threading lace: ref 9–21 s (vs 22–105)						
	Unadjusted	1.01 (0.95, 1.08)	0.762	0.97 (0.80, 1.16)	0.714	0.99 (0.91, 1.09)	0.892
	Adjusted	1.01 (0.96, 1.10)	0.496	0.94 (0.70, 1.25)	0.646	1.01 (0.92, 1.11)	0.894
	Peg board						
	Preferred hand: ref 15–22 s (vs 23–46)						
	Unadjusted	1.03 (0.97, 1.10)	0.377	1.07 (0.89, 1.28)	0.493	1.03 (0.94, 1.12)	0.585
	Adjusted	1.04 (0.97, 1.12)	0.248	1.00 (0.75, 1.33)	0.972	1.05 (0.95, 1.15)	0.349
	Non-preferred hand: ref 15–25 s (vs 25–62)						
	Unadjusted	1.02 (0.95, 1.09)	0.589	1.03 (0.86, 1.24)	0.761	0.93 (0.85, 1.02)	0.101
	Adjusted	1.04 (0.97, 1.11)	0.276	0.98 (0.74, 1.31)	0.911	0.94 (0.86, 1.03)	0.195

Participants with poor attention to task excluded.

Adjusted for: sex, maternal education, smoking in pregnancy, alcohol in pregnancy, maternal age and parity.

**Table 3:** Motor skills in children at age 7 years by category of prenatal lead, cadmium or mercury exposure: complete cases (*n* = 1558).

		**Prenatal blood lead**		**Prenatal blood cadmium**		**Prenatal blood mercury**	
		**OR (95% CI)**	***p***	**OR (95% CI)**	***p***	**OR (95% CI)**	***p***
**Balance**	Heel to toe: ref 15 steps (vs 1–14)						
	Unadjusted	1.08 (0.81, 1.43)	0.610	0.84 (0.69, 1.02)	0.081	1.14 (0.94, 1.40)	0.190
	Model 1^a^	0.99 (0.74, 1.33)	0.933	0.86 (0.69, 1.07)	0.169	1.05 (0.85, 1.29)	0.663
	Model 2^b^	0.98 (0.73, 1.33)	0.929	0.87 (0.70, 1.08)	0.201	1.06 (0.86, 1.31)	0.580
**Ball skills**	Beanbag: ref 7–10 throws (vs 1–3)						
	Unadjusted	0.86 (0.58, 1.23)	0.442	1.05 (0.79, 1.40)	0.751	1.12 (0.83, 1.50)	0.441
	Model 1^a^	0.88 (0.58, 1.32)	0.540	1.04 (0.76, 1.42)	0.805	1.15 (0.85, 1.56)	0.352
	Model 2^b^	0.88 (0.58, 1.33)	0.539	1.05 (0.77, 1.43)	0.778	1.16 (0.86, 1.57)	0.341
**Manual dexterity**	Threading lace: ref 9–21 s (vs 22–105)						
	Unadjusted	1.06 (0.80, 1.41)	0.676	0.88 (0.72, 1.07)	0.195	0.87 (0.72, 1.07)	0.181
	Model 1^a^	1.12 (0.83, 1.50)	0.468	0.85 (0.68, 1.05)	0.129	0.89 (0.72, 1.10)	0.293
	Model 2^b^	1.12 (0.83, 1.50)	0.470	0.84 (0.68, 1.04)	0.113	0.89 (0.72, 1.10)	0.290
	Peg board						
	Preferred hand: ref 15–22 s (vs 23–46)						
	Unadjusted	1.14 (0.86, 1.52)	0.371	0.99 (0.81, 1.21)	0.904	0.94 (0.77, 1.15)	0.519
	Model 1^a^	1.19 (0.88, 1.60)	0.256	0.95 (0.76, 1.18)	0.625	0.97 (0.78, 1.20)	0.756
	Model 2^b^	1.18 (0.88, 1.60)	0.266	0.94 (0.76, 1.17)	0.600	0.96 (0.78, 1.19)	0.709
	Non-preferred hand: ref 15–25 s (vs 25–62)						
	Unadjusted	1.06 (0.80, 1.41)	0.678	0.95 (0.78, 1.16)	0.595	0.82 (0.67, 1.00)	0.053
	Model 1^a^	1.14 (0.85, 1.54)	0.372	0.91 (0.74, 1.13)	0.403	0.85 (0.69, 1.05)	0.122
	Model 2^b^	1.13 (0.84, 1.52)	0.406	0.92 (0.74, 1.14)	0.428	0.84 (0.68, 1.04)	0.114

Reference categories: lead < 5.00 µg/dl, cadmium < 0.25 µg/l, mercury < 2.00 µg/l.

Participants with poor attention to task excluded.

a Model 1 adjusted for: sex, maternal education, smoking in pregnancy, alcohol in pregnancy, maternal age and parity.

b Model 2 adjusted for: sex, maternal education, smoking in pregnancy, alcohol in pregnancy, maternal age, parity and gestational age at prenatal blood sampling.

**Table 4:** Motor skills in child at age 7 years by category of prenatal lead, cadmium or mercury exposure: all cases.

		**Prenatal blood lead (µg/dl)**			**Prenatal blood cadmium (µg/l)**			**Prenatal blood mercury (µg/l)**		
		**< 5**	**≥ 5**	***p***	**< 0.26**	**> 0.26**	***p***	**< 1.99**	**> 1.99**	***p***
**Balance**	Heel to toe steps									
	15 (good)	925 (85.5%)	157 (14.5%)	0.405	561 (53.4%)	489 (46.6%)	0.010	504 (48.3%)	540 (51.7%)	0.205
	< 15	941 (86.7%)	144 (13.3%)		511 (47.8%)	558 (52.2%)		536 (51.0%)	514 (49.0%)	
**Ball skills**	Beanbag (n)									
	4–10 (good)	1587 (85.9%)	260 (14.1%)	0.906	914 (50.7%)	889 (49.3%)	0.969	874 (49.1%)	905 (50.9%)	0.308
	1–3	268 (86.2%)	43 (13.8%)		156 (50.8%)	151 (49.2%)		161 (52.3%)	147 (47.7%)	
**Manual dexterity**	Threading lace (s)									
	9–21 (good)	911 (85.9%)	150 (14.1%)	0.982	507 (49.1%)	525 (50.9%)	0.204	493 (47.7%)	540 (52.3%)	0.173
	22–105	804 (85.9%)	132 (14.4%)		479 (50.2%)	442 (48.0%)		456 (51.9%)	441 (55.0%)	
	Peg board (s)									
	Preferred hand									
	15–22 (good)	1049 (86.7%)	161 (13.3%)	0.215	605 (51.2%)	576 (48.8%)	0.420	568 (48.8%)	597 (51.2%)	0.508
	23–46	793 (84.8%)	142 (15.2%)		453 (49.5%)	463 (50.5%)		456 (50.2%)	452 (49.8%)	
	Non-preferred hand									
	15–25 (good)	994 (86.3%)	158 (13.7%)	0.743	581 (51.6%)	546 (48.4%)	0.333	537 (48.2%)	578 (51.8%)	0.080
	26–63	821 (85.8%)	136 (14.2%)		462 (49.4%)	473 (50.6%)		481 (52.1%)	443 (47.9%)	

Manual dexterity variables categorised on median.

Participants with poor task attention excluded.

**Table 5:** Association of motor skills with prenatal blood lead, cadmium or mercury exposure: all cases.

		**Prenatal blood lead**		**Prenatal blood cadmium**		**Prenatal blood mercury**	
		**OR (95% CI)**	***p***	**OR (95% CI)**	***p***	**OR (95% CI)**	***p***
**Balance**	Heel to toe: ref 15 steps (vs 1–14)						
	Unadjusted	1.11 (0.87, 1.42)	0.405	0.80 (0.67, 0.95)	0.010	1.18 (0.94, 1.33)	0.205
	Adjusted	1.03 (0.78, 1.36)	0.818	0.82 (0.67, 1.01)	0.059	1.08 (0.89, 1.31)	0.458
**Ball skills**	Beanbag: ref 4–10 throws (vs 1–3)						
	Unadjusted	1.08 (0.84, 1.38)	0.557	1.11 (0.94, 1.32)	0.220	0.99 (0.83, 1.18)	0.910
	Adjusted	1.11 (0.84, 1.46)	0.467	1.16 (0.94, 1.42)	0.159	1.13 (0.93, 1.38)	0.227
**Manual dexterity**	Threading lace: ref 9–21 s (vs 22–105)						
	Unadjusted	1.00 (0.78, 1.28)	0.982	0.89 (0.75, 1.06)	0.204	0.88 (0.74, 1.06)	0.173
	Adjusted	1.04 (0.78, 1.38)	0.784	0.92 (0.74, 1.13)	0.413	0.91 (0.74, 1.11)	0.343
	Peg board (s)						
	Preferred hand: ref 15–22 (vs 23–46)						
	Unadjusted	1.17 (0.91, 1.50)	0.215	1.07 (0.90, 1.28)	0.420	0.94 (0.79, 1.12)	0.943
	Adjusted	1.25 (0.95, 1.65)	0.997	1.00 (0.81, 1.22)	0.907	0.97 (0.80, 1.19)	0.771
	Non-preferred hand: ref 15–25 (vs 25–62)						
	Unadjusted	1.01 (0.81, 1.33)	0.743	1.09 (0.92, 1.30)	0.333	0.86 (0.72, 1.02)	0.080
	Adjusted	1.13 (0.86, 1.49)	0.394	0.97 (0.79, 1.12)	0.742	0.83 (0.68, 1.02)	0.071

Reference categories: lead < 5 µg/dl, cadmium < 0.26 µg/l, mercury < 1.99 µg/l.

Participants with poor attention excluded.

Adjusted for: sex, maternal education, smoking in pregnancy, alcohol in pregnancy, maternal age and parity.

**Table 6:** Associations of motor skills with quartiles of prenatal blood lead, cadmium or mercury exposure.

			**Prenatal blood lead**		**Prenatal blood cadmium**		**Prenatal blood mercury**	
			**OR (95% CI)**	***p***	**OR (95% CI)**	***p***	**OR (95% CI)**	***p***
**Balance**	Heel to toe: ref 15 steps (vs 1–14): ref Q1	Q4	1.06 (0.81, 1.39)	0.691	0.83 (0.61, 1.12)	0.224	1.05 (0.79, 1.39)	0.718
**Ball skills**	Beanbag: ref 4–10 throws (vs 1–3): ref Q1	Q4	0.92 (0.70, 1.21)	0.535	0.99 (0.72, 1.35)	0.939	1.13 (0.85, 1.49)	0.410
**Manual dexterity**	Threading lace: ref 9–21 s (vs 22–105): ref Q1	Q4	0.93 (0.70, 1.24)	0.634	0.95 (0.69, 1.30)	0.946	1.21 (0.90, 1.61)	0.206
	Peg board							
	Preferred hand: ref 15–22 s (vs 23–46): ref Q1	Q4	1.23 (0.94, 1.63)	0.138	1.04 (0.76, 1.42)	0.915	1.11 (0.84, 1.48)	0.460
	Non-preferred hand: ref 15–25 (vs 25–62 s): ref Q1	Q4	1.04 (0.79, 1.36)	0.792	1.06 (0.78, 1.45)	0.721	0.78 (0.59, 1.03)	0.083

Reference categories: lead < 5 µg/dl, cadmium < 0.25 µg/l, mercury < 2.00 µg/l.

Participants with poor attention excluded.

Adjusted for: sex, maternal education, smoking in pregnancy, alcohol in pregnancy, maternal age and parity.
