# Supplementary material for: The ALSPAC Coordination Test (subtests of the Movement ABC): Methodology and data on associations with prenatal exposures to lead, cadmium and mercury
Source: Data Brief. 2018 May 5;19:189–97. doi: 10.1016/j.dib.2018.04.130 (PMC5993154; doi:10.1016/j.dib.2018.04.130)
Supplement: Supplementary file 1 — Supplementary material [file mmc1.docx]

**Completing financial interests**

The authors have no competing interests.
